# Hypertension and Racial/Ethnic Disparities in Sleep Outcomes among Adults in the 2011–2023 National Health and Nutrition Examination Survey

**DOI:** 10.1093/ajh/hpaf110

**Published:** 2025-08-22

**Authors:** Faith E Metlock, Oluwabunmi Ogungbe, Ketum Ateh Stanislas, Xiaoyue Liu, Thomas Hinneh, Ruth-Alma N Turkson-Ocran, Binu Koirala, Cheryl R Himmelfarb, Yvonne Commodore-Mensah

**Affiliations:** Johns Hopkins School of Nursing, Baltimore, MD, USA; Johns Hopkins School of Nursing, Baltimore, MD, USA; Johns Hopkins Bloomberg School of Public Health, Baltimore, MD, USA; Johns Hopkins School of Nursing, Baltimore, MD, USA; NYU Rory Meyers College of Nursing, New York, NY, USA; Johns Hopkins School of Nursing, Baltimore, MD, USA; Division of General Medicine, Beth Israel Deaconess Medical Center, Boston, MA, USA; Harvard Medical School, Boston, MA, USA; Johns Hopkins School of Nursing, Baltimore, MD, USA; Johns Hopkins School of Nursing, Baltimore, MD, USA; Johns Hopkins Bloomberg School of Public Health, Baltimore, MD, USA; Johns Hopkins School of Nursing, Baltimore, MD, USA; Johns Hopkins Bloomberg School of Public Health, Baltimore, MD, USA

**Keywords:** blood pressure, hypertension, race/ethnicity, sleep health

## Abstract

**BACKGROUND:**

Racial/ethnic disparities in sleep outcomes may compound cardiovascular health (CVH) risks, particularly among adults with hypertension (HTN). This study examines differences in sleep health across racial/ethnic groups, with a primary focus on adults with HTN.

**METHODS:**

We analyzed NHANES data (2011–2023) for adults aged ≥20 years. Sleep outcomes included daytime sleepiness (2015–2020), sleep duration (2011–2023), and sleep quality (2011–2020). HTN was defined as blood pressure ≥130/80 mmHg, self-reported diagnosis, or antihypertensive use. Regression models assessed associations between race/ethnicity and each sleep outcome, adjusting for relevant covariates. Analyses were stratified by HTN status to examine differences among adults with and without HTN. All models incorporated NHANES sampling weights and accounted for the complex survey design.

**RESULTS:**

Among ~201.7 million US adults (mean age: 48.0 ± 17.1 years), 52.6% had HTN. Among adults with HTN, NH Black and NH Asian adults had higher odds of short sleep (<7 hours) compared to NH White adults (aOR: 1.86, 95% CI: 1.58–2.21; aOR: 1.58, 95% CI: 1.29–1.93). Odds of poor sleep quality were elevated in NH Asian (aOR: 2.45, 95% CI: 2.09–2.89), NH Black (aOR: 1.47, 95% CI: 1.29–1.67), and Mexican-American/Hispanic adults (aOR: 1.57, 95% CI: 1.34–1.83). In contrast, excessive daytime sleepiness was less common among NH Asian (aOR: 0.17, 95% CI: 0.11–0.25), NH Black (aOR: 0.49, 95% CI: 0.34–0.72), and Hispanic adults (aOR: 0.38, 95% CI: 0.27–0.53) than NH White adults.

**CONCLUSIONS:**

Racial/ethnic disparities in sleep health are more pronounced among adults with HTN, compounding their overall cardiovascular health risk.

## Graphical Abstract

**Graphical Abstract:**
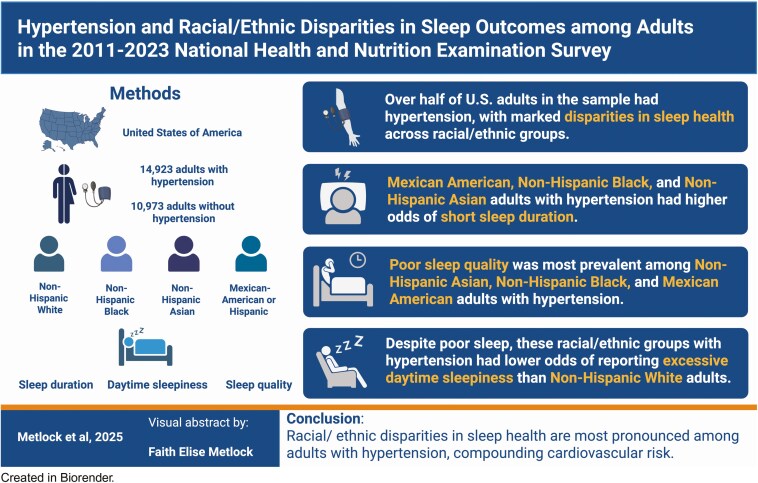

Racial/ethnic disparities in hypertension (HTN) remain a critical public health issue in the United States, with Non-Hispanic (NH) Black, Hispanic, and NH Asian adults experiencing disproportionately higher prevalence and lower control rates compared to NH White adults. NH Black adults, in particular, have the highest HTN prevalence and lowest rates of control.^1^ These disparities, driven by socioeconomic and structural inequities, underscore the need for a deeper understanding of factors contributing to health inequities.^2–5^ Emerging evidence suggests that sleep health—an essential component of overall health—may be a significant, yet underexplored, contributor to these disparities.^6,7^

Sleep health encompasses several dimensions, including sleep duration, quality, and excessive daytime sleepiness (EDS), which have well-documented relationships with cardiometabolic outcomes, including HTN and cardiovascular disease.^6^ Both short and long sleep durations have been linked to increased cardiovascular risk, with the American Academy of Sleep Medicine recommending 7–9 hours per night for optimal health.^7,8^ Poor sleep quality, characterized by disturbances such as difficulty falling or staying asleep, has similarly been associated with adverse health outcomes independent of sleep duration.^9^ EDS, marked by frequent episodes of involuntary sleep or impaired alertness during waking hours, is particularly prevalent among individuals with HTN and further exacerbates cardiovascular risks.^10,11^

There has been an increase in research examining sleep and CVH following the American Heart Association’s recognition of sleep as a vital component in the Life’s Essential 8 framework.^12^ Despite this growing recognition, there remains a dearth of research exploring how sleep outcomes differ among adults from varying racial/ethnic backgrounds. To address these gaps, this study examines racial and ethnic differences in sleep health outcomes—duration, quality, and daytime sleepiness—among US adults using data from the National Health and Nutrition Examination Survey (NHANES). By including both individuals with and without HTN, we aim to explore how sleep patterns differ across racial and ethnic groups and assess whether these differences persist independent of HTN status. Understanding these variations is imperative for identifying targeted strategies to improve sleep health and reduce health disparities in cardiovascular outcomes.

## METHODS

### Study Design and Population

**Figure 1.**
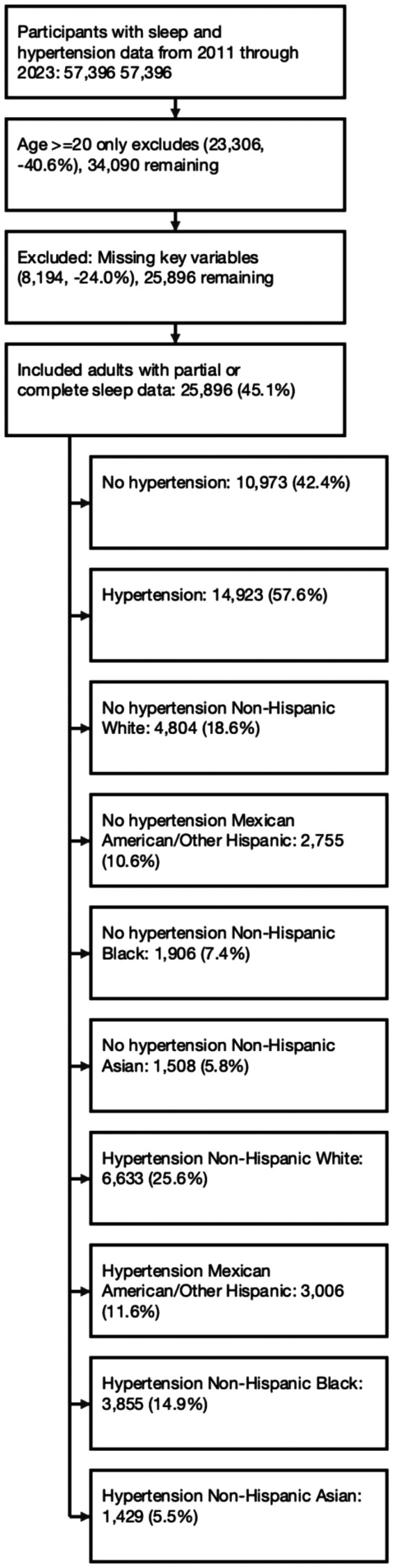
Flowchart of study sample selection. This CONSORT-style diagram outlines the inclusion and exclusion criteria used to construct the analytic samples from the 2011–2023 National Health and Nutrition Examination Survey (NHANES) cycles. Adults aged 20 years and older were included if they had complete data on sleep outcomes, race/ethnicity, and relevant covariates. Final analytic sample sizes varied by sleep measure based on availability across NHANES cycles: 25,896 participants were included in the sleep duration analysis (2011–2023), 21,705 participants were included in the sleep quality analysis (2011–2020), and 12,364 participants were included in the daytime sleepiness analysis (2015–2020). All analyses applied appropriate NHANES sampling weights and accounted for the complex survey design.

We conducted a cross-sectional analysis using National Health and Nutrition Examination Survey (NHANES) data from 2011 to 2023, a comprehensive research program conducted that gathers health information from a representative sample of the non-institutionalized civilian population in the USA. The survey employs a complex, multistage sampling design to ensure the generalizability of its findings to the broader US population. Adults aged 20 years and older with available sleep and HTN data were included. Sleep-related variables varied by cycle: daytime sleepiness (2015–2020), sleep duration (2011–2023), and sleep quality (2011–2020).^13^ Of the initial 57,396 participants, 35,816 remained after excluding those under 20 years, and 25,896 with complete data were included in the final analysis. Among these, 14,923 had HTN, while 10,973 did not. Among adults with HTN, the racial/ethnic distribution included 6,633 NH White, 3,006 Mexican-American/Other Hispanic, 3,855 NH Black, and 1,429 NH Asian adults. Among adults without HTN, the racial/ethnic distribution included 4,804 NH White, 2,755 Mexican-American/Other Hispanic, 1,906 NH Black, and 1,508 NH Asian adults (**Figure 1**).

### Definition of HTN

Participants were classified as having HTN based on the following criteria: if they answered “yes” to the question “Have you ever been diagnosed with HTN or high blood pressure by a healthcare professional?”; if they self-reported using antihypertensive medication; or if they had elevated biological measurements (systolic blood pressure (BP) ≥130 mm Hg and/or diastolic BP ≥80 mm Hg). Blood pressure was measured three times consecutively, with additional measurements taken if any of the initial readings were interrupted or incomplete. Both mean systolic BP and mean diastolic BP were defined as the average of the first three measurements.

### Sleep Outcomes

Sleep outcome variables were assessed using self-report questionnaires administered during NHANES interviews. These variables included daytime sleepiness, sleep duration, and sleep quality. Daytime sleepiness was assessed by asking participants, “In the past month, how often do you feel overly sleepy during the day?.” The possible responses included “Never,” “Rarely (1 time a month),” “Sometimes (2–4 times a month),” “Often (5–15 times a month),” and “Almost Always (16–30 times a month).” These responses were grouped into three categories: no daytime sleepiness (never), mild daytime sleepiness (rarely/sometimes), and EDS (often/always). This classification aligns with previous studies.^14,15^ Sleep duration was captured through a single question: “How much sleep do you usually get at night on weekdays or workdays?” Responses were categorized as recommended (7–9 hours), short (<7 hours), or long (>9 hours) based on prior research and established guidelines.^7^ Sleep quality was evaluated based on the question, “Ever told doctor had trouble sleeping?” and categorized based on responses as either good or poor. Good sleep quality was defined as self-reported having no trouble sleeping, while poor sleep quality was defined as self-reported having trouble sleeping.

### Covariates

Covariates included in all adjusted models were selected based on previous literature and theoretical relevance to both sleep and HTN. These included age, sex (male vs. female), education level (<high school, high school–some college, college or above), poverty-income ratio (PIR <1 vs. ≥1), marital status (married vs. unmarried), health insurance status (insured vs. uninsured), employment status (employed vs. unemployed), smoking status (never, former, current), body mass index (BMI category: normal, overweight, obese), diabetes, and chronic kidney disease (CKD). Variables were recoded into consistent formats across cycles for harmonization.

### Statistical Analysis

Descriptive statistics were used to summarize demographic variables, health conditions, sleep outcomes, and covariates across race/ethnicity categories and by HTN status. Race/ethnicity was categorized as NH White, Mexican-American/Other Hispanic, NH Black, and NH Asian. Sleep outcomes were categorized as follows: sleep duration into short (≤6 hours), recommended (7–9 hours, reference group), and long (>9 hours); sleep quality into good (no reported trouble sleeping) and poor (self-reported trouble sleeping); and daytime sleepiness into no (never), mild (1–4 times/month), and excessive (5–30 times/month). Chi-square tests were used for categorical variables and *t*-tests or ANOVA for continuous variables. Multinomial logistic regression models were used to estimate crude and adjusted odds ratios (ORs and aORs) and 95% confidence intervals (CIs) for the categorical outcomes of sleep duration and daytime sleepiness. Binary logistic regression was used for the dichotomous outcome of sleep quality. All models were adjusted for age, sex, education, poverty-income ratio, health insurance, marital status, smoking status, diabetes, chronic kidney disease, body mass index, and employment status. Analyses were stratified by HTN status and included interaction terms for age; stratified results were presented for sleep duration and sleep quality, while no effect modification by age was observed for daytime sleepiness.

### Weighting Methodology for NHANES Analysis

To account for the complex survey design when analyzing multiple NHANES cycles with varying sleep parameter availability, we applied cycle-specific sampling weights adjusted proportionally to their respective time periods. Tables 1–3 and Supplementary Tables S1–S6 were weighted for the entire sample using combined data from all cycles (2011–2023) to provide a comprehensive overview of descriptive characteristics of the study population with weights proportioned as 2/11.2 for each standard 2-year cycle and 3.2/11.2 for the 2017–2020 period. For outcome-specific analyses, we created separate weighting schemes: daytime sleepiness analyses (2015–2020) combined 2015–2016 and 2017 March 2020 weights with proportions of 2/5 and 3/5 respectively; sleep quality analyses (2011–2020) adjusted weights from four survey cycles by 2/9 for each standard two-year cycle (2011–2016) and 3/9 for the extended 2017–2020 cycle; and sleep duration analyses (2011–2023) incorporated all available cycles with weights proportioned as 2/11.2 for each standard 2-year cycle and 3.2/11.2 for the 2017–2020 period. All analyses maintained the complex survey design using primary sampling units and stratification variables, with appropriate subpopulation approaches to ensure correct variance estimation. Statistical analyses were conducted using STATA version 18.0.^16^ A *P*-value <0.05 was defined as statistically significant. The data underlying this article are available in the National Health and Nutrition Examination Survey (NHANES), conducted by the National Center for Health Statistics (NCHS), Centers for Disease Control and Prevention (CDC), and publicly available at: https://www.cdc.gov/nchs/nhanes/index.htm

## RESULTS

### Sociodemographic Characteristics by Race/Ethnicity and HTN Status

Of approximately 201.7 million US adults, 52.6% had HTN (~106.2 million). Adults with HTN were older (mean age: 54.4 ± 16.0 years) and most were NH White (68.7%), followed by NH Black (13.3%), Mexican-American/Other Hispanic (13.0%), and NH Asian (5.0%). Most had less than a college degree (71.9%), were overweight/obese (80.6%), employed (56.1%), and insured (88.4%). NH Asian adults had the highest rates of college completion (47.9%) and the lowest rates of obesity (55.1%) and diagnosed sleep disorders (4.2%). NH Black adults had the highest prevalence of current smokers (23.4%) and diabetes (19.6%). Mexican-American/Other Hispanic adults were most likely to be uninsured (27.1%) and have less than a high school education (61.4%). Among adults with HTN, 35.1% reported recommended sleep duration, 27.8% short sleep, and 37.2% long sleep (**Table 1**). Poor sleep quality (Supplementary Table S1) was reported by 65.7%, mild daytime sleepiness by 27.8%, and EDS by 37.2% (Supplementary Table S2). Among an estimated 95.5 million adults without HTN, individuals were younger compared to those with HTN (mean age: 40.8 ± 15.2 vs. 54.4 ± 16.0), more often employed (72.2% vs. 56.1%) and college educated (38.0% vs. 28.1%), and had lower rates of obesity (61.9% vs. 80.6%) and diabetes (4.0% vs. 16.5%). NH White adults remained the majority (67.8%), followed by Mexican-American/Other Hispanic (15.2%), NH Black (11.3%), and NH Asian (5.7%). Further, adults without HTN were more likely to report recommended sleep (38.1% vs. 35.1%) (Supplementary Table S3) and poor sleep quality (76.6.3% vs. 65.7%) (Supplementary Table S4). EDS was comparable between groups (27.1% vs. 28.0%) (Supplementary Table S5).

**Table 1.** Sociodemographic characteristics by race/ethnicity among adults with hypertension (2011–2023, sleep duration analysis)

	Non-Hispanic White	Mexican-American/Other Hispanic	Non-Hispanic Black	Non-Hispanic Asian	Total	*P*-value
Sample size	(*N* = 6,633)	(*N* = 3,006)	(*N* = 3,855)	(*N* = 1,429)	(*N* = 14,923)	
Population size	72,333,829	13,777,928	14,148,147	5,334,775	106,194,679	
Age						
Mean (SD)	56.21 ± (15.90)	48.95 ± (15.47)	51.34 ± (15.57)	51.98 ± (15.75)	54.40 ± (16.03)	<0.001
Sleep duration						
Recommended	26,931,790 36.93%	4,687,187 34.02%	3,826,695 27.05%	1,784,304 33.45%	37,229,976 35.06%	<0.001
Short	18,048,537 24.75%	4,236,265 30.75%	5,590,705 39.52%	1,606,751 30.12%	29,482,257 27.76%	
Long	27,953,502 38.33%	4,854,476 35.23%	4,730,747 33.44%	1,943,720 36.43%	39,482,446 37.18%	
Ever told by doctor have sleep disorder						
No	22,705,923 85.83%	3,965,541 90.97%	4,464,848 88.74%	1,485,887 95.85%	32,622,199 87.24%	<0.001
Yes	3,747,228 14.17%	393,521 9.03%	566,699 11.26%	64,275 4.15%	4,771,725 12.76%	
Sex						
Male	37,339,141 51.20%	7,701,082 55.89%	6,297,233 44.51%	2,664,776 49.95%	54,002,232 50.85%	<0.001
Female	35,594,687 48.80%	6,076,846 44.11%	7,850,914 55.49%	2,669,999 50.05%	52,192,447 49.15%	
Marital status						
Unmarried	27,302,557 37.43%	5,547,995 40.27%	8,507,630 60.13%	1,439,390 26.98%	42,797,572 40.30%	<0.001
Married	45,631,272 62.57%	8,229,933 59.73%	5,640,517 39.87%	3,895,385 73.02%	63,397,108 59.70%	
Education						
<High school or less	25,574,990 35.07%	8,452,752 61.35%	6,595,887 46.62%	1,684,012 31.57%	42,307,642 39.84%	<0.001
High school–Some College	24,791,006 33.99%	3,453,925 25.07%	4,737,987 33.49%	1,097,315 20.57%	34,080,233 32.09%	
College graduate or above	22,567,833 30.94%	1,871,251 13.58%	2,814,273 19.89%	2,553,447 47.86%	29,806,804 28.07%	
Poverty-income ratio (PIR)						
Family PIR ≥1	66,088,056 90.61%	10,158,069 73.73%	10,414,066 73.61%	4,663,931 87.43%	91,324,121 86.00%	<0.001
Family PIR <1	6,845,773 9.39%	3,619,860 26.27%	3,734,081 26.39%	670,844 12.57%	14,870,558 14.00%	
Insurance						
Uninsured	5,656,953 7.76%	3,729,243 27.07%	2,364,527 16.71%	558,769 10.47%	12,309,492 11.59%	<0.001
Insured	67,276,876 92.24%	10,048,685 72.93%	11,783,620 83.29%	4,776,006 89.53%	93,885,188 88.41%	
Employment						
Unemployed	33,010,608 45.26%	5,308,985 38.53%	6,290,771 44.46%	2,065,368 38.72%	46,675,732 43.95%	<0.001
Employed	39,923,221 54.74%	8,468,943 61.47%	7,857,376 55.54%	3,269,407 61.28%	59,518,947 56.05%	
Smoking status						
Never Smoker	35,870,675 49.18%	8,412,281 61.06%	8,199,096 57.95%	3,945,557 73.96%	56,427,609 53.14%	<0.001
Former Smoker	24,543,475 33.65%	3,440,592 24.97%	2,660,740 18.81%	912,025 17.10%	31,556,833 29.72%	
Current Smoker	12,519,679 17.17%	1,925,055 13.97%	3,288,311 23.24%	477,193 8.94%	18,210,236 17.15%	
Body mass index classification						
Not Overweight/Obese	14,079,537 19.30%	1,589,553 11.54%	2,507,950 17.73%	2,395,745 44.91%	20,572,786 19.37%	<0.001
Overweight/Obese	58,854,292 80.70%	12,188,375 88.46%	11,640,197 82.27%	2,939,030 55.09%	85,621,893 80.63%	
Diabetes						
No	61,730,323 84.64%	11,100,090 80.56%	11,381,376 80.44%	4,516,740 84.67%	88,728,528 83.55%	<0.001
Yes	11,203,506 15.36%	2,677,839 19.44%	2,766,771 19.56%	818,035 15.33%	17,466,152 16.45%	
Chronic kidney disease						
No	69,939,409 95.89%	13,277,812 96.37%	13,426,858 94.90%	5,200,948 97.49%	101,845,027 95.90%	<0.001
Yes	2,994,420 4.11%	500,116 3.63%	721,289 5.10%	133,827 2.51%	4,349,652 4.10%	

Mean ± SD or *N* (%).

Sleep duration: From the question, “How much sleep do you usually get at night on weekdays or workdays?” Short: ≤6 hours; recommended: 7–9 hours; long: >9 hours.

All statistics were computed using the weighted sample to account for NHANES’ complex survey design.

*P*-values are from Kruskal-Wallis tests for continuous variables and chi-square tests for categorical variables.

Analysis restricted to adults with hypertension from the 2011–2023 NHANES sleep duration sample.

### Racial/Ethnic Differences in Sleep Outcomes among Adults with HTN

#### Daytime Sleepiness.

**Figure 2.**
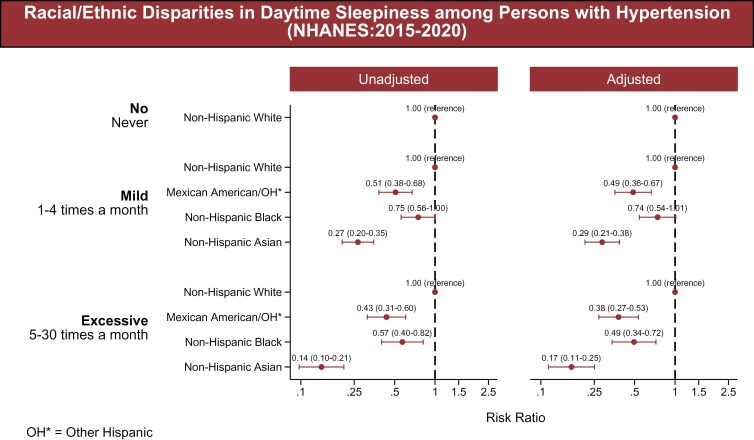
Racial/ethnic disparities in daytime sleepiness (2015–2020). The association between race/ethnicity and daytime sleepiness, categorized into three groups: No (Never), Moderate (1-4 times per month), and Excessive (5–30 times per month). The racial/ethnic groups included are NH White, Mexican-American, Other Hispanic, NH Black, and NH Asian adults. The figure presents both unadjusted and adjusted models. The associations were adjusted for age, sex, education, poverty-income ratio, health insurance, marital status, smoking status, diabetes, body mass index, chronic kidney disease, and employment status.

Mexican-American, NH Black, and NH Asian adults with HTN had lower odds of reporting both mild (rarely or sometimes) and excessive (often or almost always) levels of daytime sleepiness compared to NH White adults. Mexican-American adults had lower adjusted odds of mild sleepiness (adjusted odds ratio (aOR): 0.49, 95% confidence interval (CI): 0.36–0.67), and NH Asian adults also had lower odds (aOR: 0.29, 95% CI: 0.21–0.38). NH Black adults had lower odds as well (aOR: 0.74, 95% CI: 0.54–1.01). For excessive sleepiness, all three groups had lower adjusted odds: 0.38 (95% CI: 0.27–0.53) for Mexican-American adults, 0.49 (95% CI: 0.34–0.72) for NH Black adults, and 0.17 (95% CI: 0.11–0.25) for NH Asian adults (**Figure 2**).

#### Sleep Quality.

**Figure 3.**
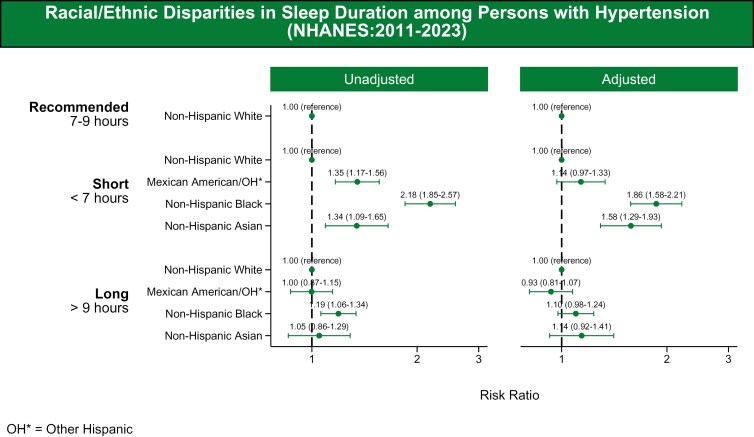
Racial/ethnic disparities in sleep duration (2011–2023). The association between race/ethnicity and sleep quality, categorized into two groups: good (self-reported as having no trouble sleeping) and poor (self-reported as having trouble sleeping). The racial/ethnic groups included are NH White, Mexican-American/Other Hispanic, NH Black, and NH Asian adults. The figure presents both unadjusted and adjusted models. The adjusted models account for covariates including age, sex, education, poverty-income ratio, health insurance, marital status, smoking status, diabetes, body mass index, chronic kidney disease, and employment status.

NH Black, Mexican-American, and NH Asian adults with HTN were more likely to report poor sleep quality (trouble sleeping) compared to NH White adults. Adjusted odds were 1.57 (95% CI: 1.34–1.83) for Mexican-American adults, 1.47 (95% CI: 1.29–1.69) for NH Black adults, and 2.45 (95% CI: 2.09–2.89) for NH Asian adults (**Figure 3**).

#### Sleep Duration.

**Figure 4.**
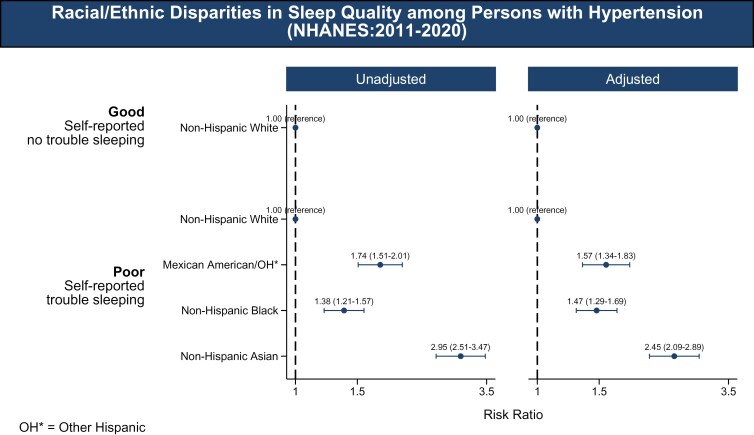
Racial/ethnic disparities in sleep quality (2011–2020). The association between race/ethnicity and sleep duration, categorized into three groups: recommended (7–9 hours), short (≤6 hours), and long (>9 hours). The racial/ethnic groups included are NH White, Mexican-American/Other Hispanic, NH Black, and NH Asian adults. The figure presents both unadjusted and adjusted models. The adjusted models account for covariates including age, sex, education, poverty-income ratio, health insurance, marital status, smoking status, diabetes, body mass index, chronic kidney disease, and employment status.

Short sleep duration (<7 hours) was more frequently reported among NH Black, NH Asian, and Mexican-American adults with HTN compared to NH White adults. Adjusted odds were 1.86 (95% CI: 1.58–2.21) for NH Black adults, 1.58 (95% CI: 1.29–1.93) for NH Asian adults, and 1.14 (95% CI: 0.97–1.33) for Mexican-American adults. For long sleep duration (>9 hours), adjusted odds were 1.10 (95% CI: 0.98–1.24) for NH Black adults, 0.93 (95% CI: 0.81–1.07) for Mexican-American adults, and 1.14 (95% CI: 0.92–1.41) for NH Asian adults (**Figure 4**).

### Racial/Ethnic Differences in Sleep Outcomes among Adults without HTN

Among adults without HTN, NH Black (aOR: 2.14, 95% CI: 1.74–2.64), NH Asian (aOR: 1.09, 95% CI: 0.91–1.31), and Mexican-American (aOR: 1.23, 95% CI: 1.02–1.47) adults had higher odds of short sleep duration (<7 hours), and odds of long sleep duration (>9 hours) were similar across all groups (Supplementary Figure S1). For poor sleep quality (trouble sleeping), NH Asian adults had the highest odds (aOR: 2.14, 95% CI: 1.71–2.68), followed by NH Black (aOR: 1.77, 95% CI: 1.47–2.15) and Mexican-American (aOR: 1.58, 95% CI: 1.32–1.90) adults (Supplementary Figure S2). NH Asian adults also had the lowest odds of reporting both mild (rarely or sometimes) and excessive (often or almost always) daytime sleepiness, and all minority racial and ethnic groups had lower adjusted odds than NH White adults for both outcomes (Supplementary Figure S3).

### Subgroup Analyses

We tested for effect modification by HTN status in the association between race/ethnicity and sleep outcomes and found no interactions (daytime sleepiness: *P* = 0.800; sleep quality: *P* = 0.530; sleep duration: *P* = 0.236). A race-by-age interaction was observed for sleep duration and quality (*P* < 0.05), but not for daytime sleepiness (*P* = 0.275); therefore, we stratified by HTN status for sleep duration and quality only. In age-stratified models, disparities were greatest among adults with HTN. NH Black and NH Asian adults with HTN had consistently higher odds of short sleep across all age groups, with the largest disparities at ages 54+ (ORs: 1.98 and 1.97). Differences in long sleep were minimal. Poor sleep quality was most elevated among NH Asian adults aged 19–33 (OR: 5.40), followed by NH Black and Hispanic adults. Among adults without HTN, NH Black adults had higher odds of short sleep across all ages (e.g., OR: 2.51 at ages 54+), and NH Asian adults showed increased odds of long sleep and poor sleep quality in later adulthood (ORs: 1.46 and 2.72). Sleep quality disparities among Hispanic adults were also present but lessened with age (Supplementary Table S6).

## DISCUSSION

We observed notable racial/ethnic disparities in sleep health, specifically in sleep duration, daytime sleepiness, and sleep quality, with differences most pronounced among adults with HTN. Among those with HTN, NH Black, Mexican-American, and NH Asian adults had consistently higher odds of short sleep and poor sleep quality. Additionally, all racial/ethnic groups had lower odds of EDS compared to NH White adults. Among adults without HTN, similar disparities were observed, though they were generally smaller in magnitude. These findings suggest that sleep health disparities may be amplified in the presence of HTN and underscore the need for future interventions that address the connection between sleep and CVH, especially among racially/ethnically diverse adults with HTN.

Previous literature has shown that NH White adults experience daytime sleepiness at lower rates than racial/ethnic groups.^17–19^ However, more recent studies have indicated a shift, with racial/ethnic groups now reporting lower rates of daytime sleepiness compared to their NH White counterparts. For instance, the Multi-Ethnic Study of Atherosclerosis found that NH White adults were more likely to report EDS compared to NH Black and Hispanic adults.^20^ Similarly, another nationally representative study reported this trend of racial/ethnic groups being less likely to report daytime sleepiness.^21^ This variation in reporting daytime sleepiness across racial/ethnic groups may be due to differences in how the questions are asked. In NHANES, for example, participants are asked about the frequency of feeling sleepy during the day, which may affect how participants perceive and report their sleepiness. Additionally, chronic sleepiness may alter individuals’ perception, leading to underreporting of symptoms.^20^ Using validated instruments like the Epworth Sleepiness Scale^22^ may better capture the true prevalence of daytime sleepiness. However, it is equally important to develop future tools that work across cultural groups and ensure consistent interpretation of questions about sleepiness. This is especially important for populations with conditions like HTN, where correctly identifying and screening for EDS may improve CVH.

Our analysis found that all racial/ethnic groups, including NH Black, NH Asian, and Mexican-American/Hispanic adults, had higher odds of short sleep duration compared with NH White adults, even after adjusting for sociodemographic covariates. These findings are consistent with previous studies linking short sleep duration with increased health risks, including HTN.^23^ For example, a prior study among US adults reported that NH Black adults had an overall age-adjusted prevalence of short sleep duration that was nine percentage points higher than that of NH White adults, with variations in prevalence depending on industry and occupation.^24^ Our findings also showed that all racial/ethnic groups, including NH Black, NH Asian, and Mexican-American/Hispanic adults, had higher odds of poor sleep quality compared with NH White adults. These results are consistent with sleep literature that indicates African-American and Latino groups generally experience poorer sleep quality compared to NH White adults.^9^ A comprehensive review of racial sleep disparities similarly reported that Asian-American adults have lower sleep quality compared to NH White adults.^25^ However, sleep literature on Asian-Americans is particularly sparse, and few studies have explored intra-ethnic variations among the different subgroups within the Asian population. This limits the generalizability of our findings and highlights the need for further research within the NH Asian population, as well as the importance of intra-ethnic exploration among NH Black and Hispanic populations to better understand sleep disparities across all racial/ethnic groups.

Racial/ethnic differences in sleep health are influenced by a multitude of social and psychosocial factors, including socioeconomic disparities, occupational stress, and chronic health conditions. Even after adjusting for some of these social risk factors in our analysis, racial/ethnic disparities persisted. Workers of color, often employed in shift work and lower-wage jobs,^26,27^ may face sleep disruptions due to workplace discrimination and limited job control.^28^ Additionally, psychosocial trauma, microaggressions, and stereotype threats further impair sleep, with these racialized stressors compounding sleep disparities over time.^29^ Racial/ethnic groups are also more likely to live in socially deprived areas, shaped by redlining and segregation,^30^ where environmental factors like loud noise, bright lights, and air pollution, can disrupt sleep and increase stress.^31–33^ These disparities are even more pronounced for those lacking stable living conditions, such as refugees and individuals experiencing homelessness.^34^ Aversive racism in healthcare can also add to sleep disparities, as racial and ethnic groups may be less likely to receive sleep evaluations due to provider biases.^31–33^ Despite recommendations from the American Academy of Sleep Medicine for routine screening,^35^ many clinicians fail to ask about sleep, and patients often do not report symptoms.^36^ Moreover, racial/ethnic groups are more likely to experience multimorbidity, which exacerbates sleep disruptions. For example, sleep apnea, a condition characterized by repeated interruptions in breathing during sleep, is an important contributor to poor sleep health. Evidence suggests that sleep apnea disproportionately affects racial/ethnic minorities, with NH Black adults experiencing higher prevalence and more severe cases compared to NH White adults. This disparity may further exacerbate cardiovascular risks associated with poor sleep quality and short sleep duration.^35,36^ Given the bi-directional relationship between sleep and chronic conditions—where poor sleep influences disease and these same conditions disrupt sleep—it is plausible that HTN and other comorbidities in these populations further contribute to the sleep disparities observed.^37^

Further research is needed to understand the mechanisms driving sleep disparities, particularly in adults with HTN. This includes examining racism and discrimination as contributors to chronic stress and leveraging multi-level study designs to explore causal pathways. Future studies should also measure factors like acculturation and employ cumulative social risk measures to fully assess the influences on sleep health.^38^ Developing tools that are both valid and reliable across racial/ethnic groups, adaptable to various modalities, and culturally translatable to capture population-level differences is essential. Alongside these advancements, workplace policies that offer flexibility, such as limiting shift work, can help mitigate sleep disparities by reducing the burden on disadvantaged populations. Expanding research methodologies and improving early detection—particularly among hypertensive adults—will be key to advancing sleep health equity.^37^ These efforts can deepen understanding of sleep disparities and guide targeted interventions to improve health outcomes for those most vulnerable.

## LIMITATIONS

There are limitations to acknowledge in this study. First, the use of self-reported sleep outcomes may introduce reporting biases, whereas objective measures (e.g., sleep stages, number of awakenings during the night) through digital tools may offer a more accurate picture of sleep health, particularly in patients with HTN. Second, the cross-sectional design limits the ability to infer causality between sleep outcomes and HTN. Third, the study did not account for work schedules, such as night or rotating shifts, which are known to affect sleep patterns and could act as confounders. Including this data would offer a more complete understanding of the factors influencing sleep among racial and ethnic groups. Despite these limitations, the study has several notable strengths. The use of a large, representative sample allows for more generalizable findings across the US population. Our findings contribute valuable insights into how sleep behaviors may differentially impact HTN management and outcomes across diverse groups.

## CONCLUSION

Our study highlights that racial/ethnic disparities in sleep health, including sleep duration, daytime sleepiness, and sleep quality, are more pronounced among adults with HTN. These disparities suggest that HTN may intensify the impact of poor sleep health, compounding cardiovascular disease risk in already vulnerable populations. Addressing these inequities requires tailored, multifaceted interventions that prioritize sleep health in HTN management. This includes incorporating routine sleep assessments into HTN care, advancing culturally informed strategies, and conducting research to understand the social and structural drivers of these disparities. Focused efforts to improve sleep health among racially/ethnically minoritized adults with HTN may help reduce cardiovascular disease risk and address persistent disparities.

## Supplementary Data

Supplementary materials are available at *American Journal of Hypertension* (http://ajh.oxfordjournals.org).

hpaf110_Supplementary_Tables_S1-S6_Figures_S1-S3
