# Supplementary material for: Hypertension and Racial/Ethnic Disparities in Sleep Outcomes among Adults in the 2011–2023 National Health and Nutrition Examination Survey
Source: Am J Hypertens. 2025 Aug 22;39(1):88–97. doi: 10.1093/ajh/hpaf110 (PMC12802884; doi:10.1093/ajh/hpaf110)
Supplement: hpaf110_Supplementary_Tables_S1-S6_Figures_S1-S3 [file hpaf110_supplementary_tables_s1-s6_figures_s1-s3.docx]

**Supplemental**

**Tables**

| **Table S1. Sociodemographic Characteristics by Race/Ethnicity among Adults with Hypertension**  **(2011-2020, Sleep Quality Analysis)** | | | | | | |
| --- | --- | --- | --- | --- | --- | --- |
|  | **Non-Hispanic White** | **Mexican American/Other Hispanic** | **Non-Hispanic Black** | **Non-Hispanic Asian** | **Total** | **P-value** |
| Sample size | (N=4,803) | (N=2,575) | (N=3,434) | (N=1,285) | (N=12,097) |  |
| Population size | 73,841,449 | 13,453,710 | 14,190,166 | 5,285,853 | 106,771,177 |  |
| **Age** | | | | | | |
| Mean (SD) | 56.07 ± (15.91) | 48.66 ± (15.47) | 51.21 ± (15.48) | 52.27 ± (15.71) | 54.30 ± (16.03) | <0.001 |
| **Sleep quality** | | | | | | |
| Good | 27,896,059 37.78% | 3,476,315  25.84% | 4,344,400 30.63% | 901,571 17.06% | 36,618,345 34.30% | <0.001 |
| Poor | 45,935,924 62.22% | 9,977,395  74.16% | 9,836,833 69.37% | 4,384,282 82.94% | 70,134,434 65.70% |  |
| **Ever told by doctor have sleep disorder** | | | | | | |
| No | 28,256,259 85.83% | 4,934,895  90.97% | 5,556,256 88.74% | 1,849,103 95.85% | 40,596,514 87.24% | <0.001 |
| Yes | 4,663,217 14.17% | 489,716  9.03% | 705,226 11.26% | 79,987  4.15% | 5,938,146 12.76% |  |
| **Sex** | | | | | | |
| Male | 37,829,439 51.23% | 7,511,120  55.83% | 6,306,255 44.44% | 2,639,132 49.93% | 54,285,946 50.84% | <0.001 |
| Female | 36,012,009 48.77% | 5,942,590  44.17% | 7,883,911 55.56% | 2,646,721 50.07% | 52,485,232 49.16% |  |
| **Marital Status** | | | | | | |
| Unmarried | 27,841,506 37.70% | 5,703,395  42.39% | 8,578,809 60.46% | 1,406,696 26.61% | 43,530,406 40.77% | <0.001 |
| Married | 45,999,943 62.30% | 7,750,315  57.61% | 5,611,357 39.54% | 3,879,157 73.39% | 63,240,772 59.23% |  |
| **Education** | | | | | | |
| < High school or less | 25,895,415 35.07% | 8,289,772  61.62% | 6,707,932 47.27% | 1,673,528 31.66% | 42,566,648 39.87% | <0.001 |
| High school - some College | 25,573,562 34.63% | 3,412,162  25.36% | 4,732,361 33.35% | 1,058,681 20.03% | 34,776,767 32.57% |  |
| College graduate or above | 22,372,471 30.30% | 1,751,775  13.02% | 2,749,873 19.38% | 2,553,643 48.31% | 29,427,762 27.56% |  |
| **Family Poverty Income Ratio (PIR)** | | | | | | |
| Family PIR ≥ 1 | 66,880,715 90.57% | 9,776,625  72.67% | 10,344,880 72.90% | 4,625,271 87.50% | 91,627,491 85.82% | <0.001 |
| Family PIR <1 | 6,960,733 9.43% | 3,677,085  27.33% | 3,845,286 27.10% | 660,582 12.50% | 15,143,686 14.18% |  |
| **Insurance** | | | | | | |
| Uninsured | 6,275,424 8.50% | 3,770,779  28.03% | 2,583,743 18.21% | 602,004 11.39% | 13,231,949 12.39% | <0.001 |
| Insured | 67,566,025 91.50% | 9,682,931  71.97% | 11,606,424 81.79% | 4,683,849 88.61% | 93,539,229 87.61% |  |
| **Employment** | | | | | | |
| Unemployed | 33,229,639 45.00% | 5,132,958  38.15% | 6,330,189 44.61% | 2,093,913 39.61% | 46,786,700 43.82% | <0.001 |
| Employed | 40,611,809 55.00% | 8,320,752  61.85% | 7,859,977 55.39% | 3,191,940 60.39% | 59,984,478 56.18% |  |
| **Smoking status** | | | | | | |
| Never Smoker | 35,728,487 48.39% | 8,052,206  59.85% | 8,097,806 57.07% | 3,959,220 74.90% | 55,837,719 52.30% | <0.001 |
| Former Smoker | 25,142,032 34.05% | 3,451,672  25.66% | 2,697,078 19.01% | 875,298 16.56% | 32,166,080 30.13% |  |
| Current Smoker | 12,970,930 17.57% | 1,949,831  14.49% | 3,395,282 23.93% | 451,335 8.54% | 18,767,379 17.58% |  |
| **Body Mass Index Classification** | | | | | | |
| Not Overweight/Obese | 14,170,183 19.19% | 1,532,422  11.39% | 2,641,883 18.62% | 2,459,299 46.53% | 20,803,787 19.48% | <0.001 |
| Overweight/Obese | 59,671,265 80.81% | 11,921,288 88.61% | 11,548,284 81.38% | 2,826,554 53.47% | 85,967,390 80.52% |  |
| **Diabetes** | | | | | | |
| No | 62,543,028 84.70% | 10,869,579 80.79% | 11,507,501 81.09% | 4,442,489 84.04% | 89,362,598 83.70% | <0.001 |
| Yes | 11,298,421 15.30% | 2,584,130 19.21% | 2,682,665 18.91% | 843,364 15.96% | 17,408,580 16.30% |  |
| **Chronic Kidney Disease** | | | | | | |
| No | 70,924,645 96.05% | 12,943,229 96.21% | 13,418,744 94.56% | 5,139,988 97.24% | 102,426,606 95.93% | <0.001 |
| Yes | 2,916,804 3.95% | 510,481  3.79% | 771,423  5.44% | 145,865 2.76% | 4,344,572 4.07% |  |
| Mean ± SD or N (%).  Sleep quality from the question, “Ever told doctor had trouble sleeping?” Good: No trouble sleeping; Poor: Trouble sleeping  All statistics were computed using the weighted sample to account for NHANES’ complex survey design.  P-values are from Kruskal-Wallis tests for continuous variables and chi-square tests for categorical variables.  Analysis restricted to adults with hypertension from the 2011–2020 NHANES sleep duration sample. | | | | | | |

| **Table S2. Sociodemographic Characteristics by Race/Ethnicity among Adults with Hypertension**  **(2015-2020, Daytime Sleepiness Analysis)** | | | | | | |  |
| --- | --- | --- | --- | --- | --- | --- | --- |
|  | **Non-Hispanic White** | **Mexican American/Other Hispanic** | **Non-Hispanic Black** | **Non-Hispanic Asian** | **Total** | **P-value** |  |
| Sample size | (N=2,544) | (N=1,576) | (N=1,954) | (N=764) | (N=6,838) |  |  |
| Population szie | 73,463,273 | 14,422,864 | 14,271,632 | 6,042,172 | 108,199,940 |  | |
| **Age** | | | | | | |  |
| Mean (SD) | 56.09 ±  (16.08) | 49.05 ±  (15.35) | 51.48 ±  (15.49) | 52.01 ± (15.87) | 54.32 ± (16.12) | <0.001 |  |
| **Daytime Sleepiness** | | | | | | |  |
| No | 26,931,790 36.93% | 4,687,187  34.02% | 3,826,695 27.05% | 1,784,304 33.45% | 37,229,976 35.06% | <0.001 |  |
| Mild | 18,048,537 24.75% | 4,236,265  30.75% | 5,590,705 39.52% | 1,606,751 30.12% | 29,482,257 27.76% |  |  |
| Excessive | 27,953,502 38.33% | 4,854,476  35.23% | 4,730,747 33.44% | 1,943,720 36.43% | 39,482,446 37.18% |  |  |
| **Ever told by doctor have sleep disorder** | | | | | | |  |
| No | 0 .% | 0 .% | 0 .% | 0 .% | 0 .% | <0.001 |  |
| Yes | 0 .% | 0 .% | 0 .% | 0 .% | 0 .% |  |  |
| **Sex** | | | | | | |  |
| Male | 37,447,810 50.97% | 8,234,576  57.09% | 6,206,901 43.49% | 2,942,981 48.71% | 54,832,268 50.68% | <0.001 |  |
| Female | 36,015,463 49.03% | 6,188,288  42.91% | 8,064,731 56.51% | 3,099,191 51.29% | 53,367,672 49.32% |  |  |
| **Marital Status** | | | | | | |  |
| Unmarried | 26,807,401 36.49% | 5,802,366  40.23% | 8,117,411 56.88% | 1,540,194 25.49% | 42,267,371 39.06% | <0.001 |  |
| Married | 46,655,872 63.51% | 8,620,498  59.77% | 6,154,222 43.12% | 4,501,978 74.51% | 65,932,569 60.94% |  |  |
| **Education** | | | | | | |  |
| < High school or less | 25,996,742 35.39% | 8,356,391  57.94% | 6,382,393 44.72% | 1,944,675 32.19% | 42,680,201 39.45% | <0.001 |  |
| High school - some College | 25,806,278 35.13% | 3,862,786  26.78% | 4,788,239 33.55% | 1,144,305 18.94% | 35,601,608 32.90% |  |  |
| College graduate or above | 21,660,252 29.48% | 2,203,687  15.28% | 3,101,000 21.73% | 2,953,192 48.88% | 29,918,131 27.65% |  |  |
| **Poverty Income Ratio (PIR)** | | | | | | |  |
| Family PIR ≥ 1 | 67,530,211 91.92% | 10,820,795 75.03% | 10,791,159 75.61% | 5,308,006 87.85% | 94,450,171 87.29% | <0.001 |  |
| Family PIR <1 | 5,933,062 8.08% | 3,602,068  24.97% | 3,480,473 24.39% | 734,166 12.15% | 13,749,770 12.71% |  |  |
| **Insurance** | | | | | | |  |
| Uninsured | 4,893,435 6.66% | 3,368,905  23.36% | 2,347,513 16.45% | 564,134 9.34% | 11,173,987 10.33% | <0.001 |  |
| Insured | 68,569,838 93.34% | 11,053,959 76.64% | 11,924,119 83.55% | 5,478,038 90.66% | 97,025,953 89.67% |  |  |
| **Employment** | | | | | | |  |
| Unemployed | 31,981,567 43.53% | 5,292,796  36.70% | 5,854,016 41.02% | 2,331,994 38.60% | 45,460,373 42.02% | 0.016 |  |
| Employed | 41,481,706 56.47% | 9,130,068  63.30% | 8,417,616 58.98% | 3,710,178 61.40% | 62,739,567 57.98% |  |  |
| **Smoking status** | | | | | | |  |
| Never Smoker | 35,616,489 48.48% | 8,740,413  60.60% | 8,210,741 57.53% | 4,680,045 77.46% | 57,247,688 52.91% | <0.001 |  |
| Former Smoker | 25,739,409 35.04% | 3,620,778  25.10% | 2,799,838 19.62% | 878,193 14.53% | 33,038,217 30.53% |  |  |
| Current Smoker | 12,107,375 16.48% | 2,061,673  14.29% | 3,261,054 22.85% | 483,934 8.01% | 17,914,035 16.56% |  |  |
| **Body Mass Index Classification** | | | | | | |  |
| Not Overweight/Obese | 13,470,598 18.34% | 1,402,420  9.72% | 2,568,299 18.00% | 2,797,919 46.31% | 20,239,236 18.71% | <0.001 |  |
| Overweight/Obese | 59,992,675 81.66% | 13,020,444 90.28% | 11,703,333 82.00% | 3,244,253 53.69% | 87,960,705 81.29% |  |  |
| **Diabetes** | | | | | | |  |
| No | 61,581,671 83.83% | 11,627,386 80.62% | 11,536,479 80.84% | 5,011,631 82.94% | 89,757,166 82.95% | 0.181 |  |
| Yes | 11,881,602 16.17% | 2,795,478 ]19.38% | 2,735,153 19.16% | 1,030,541 17.06% | 18,442,774 17.05% |  |  |
| **Chronic Kidney Disease** | | | | | | |  |
| No | 70,226,607 95.59% | 13,877,670 96.22% | 13,453,095 94.26% | 5,858,371 96.96% | 103,415,743 95.58% | 0.008 |  |
| Yes | 3,236,665 4.41% | 545,194  3.78% | 818,537  5.74% | 183,801 3.04% | 4,784,198 4.42% |  |  |
| Mean ± SD or N (%).  Daytime sleepiness: From the question, “In the past month, how often do you feel overly sleepy during the day?” No: Never (0 times/month); Mild: Rarely (1 time/month) or Sometimes (2–4 times/month); Excessive: Often (5–15 times/month) or Almost Always (16–30 times/month);  All statistics were computed using the weighted sample to account for NHANES’ complex survey design.  P-values are from Kruskal-Wallis tests for continuous variables and chi-square tests for categorical variables.  Analysis restricted to adults with hypertension from the 2015–2020 NHANES sleep duration sample. | | | | | | |  |
|  |  |  |  |  |  |  |  |

| **Table S3. Sociodemographic Characteristics by Hypertension Status**  **(2011-2023, Sleep Duration Analysis)** | | | | |
| --- | --- | --- | --- | --- |
|  | **No hypertension** | **Hypertension** | **Total** | **P-value** |
| Sample size | (N=10,973) | (N=14,923) | (N=25,896) |  |
| Population size | 95,482,006 | 106,194,679 | 201,676,685 |  |
| **Age** | | | | |
| Mean (SD) | 40.79 ±  (15.24) | 54.40 ±  (16.03) | 47.96 ± (17.07) | <0.001 |
| **Race/Ethnicity** | | | | |
| Non-Hispanic White | 63,797,854 66.82% | 72,933,829 68.68% | 136,731,683 67.80% | <0.001 |
| Mexican American/Other Hispanic | 16,796,052 17.59% | 13,777,928 12.97% | 30,573,980 15.16% |  |
| Non-Hispanic Black | 8,693,961  9.11% | 14,148,147 13.32% | 22,842,108 11.33% |  |
| Non-Hispanic Asian | 6,194,140  6.49% | 5,334,775 5.02% | 11,528,915 5.72% |  |
| **Sleep Duration** | | | | |
| Recommended | 36,334,377 38.05% | 37,229,976 35.06% | 73,564,353 36.48% | <0.001 |
| Short | 24,338,590 25.49% | 29,482,257 27.76% | 53,820,847 26.69% |  |
| Long | 34,809,040 36.46% | 39,482,446 37.18% | 74,291,485 36.84% |  |
| **Ever told by doctor have sleep disorder** | | | | |
| No | 32,915,804 93.15% | 32,622,199 87.24% | 65,538,002 90.11% | <0.001 |
| Yes | 2,419,040  6.85% | 4,771,725 12.76% | 7,190,765 9.89% |  |
| **Sex** | | | | |
| Male | 42,839,269 44.87% | 54,002,232 50.85% | 96,841,502 48.02% | <0.001 |
| Female | 52,642,737 55.13% | 52,192,447 49.15% | 104,835,184 51.98% |  |
| **Marital status** | | | | |
| Unmarried | 40,632,707 42.56% | 42,797,572 40.30% | 83,430,279 41.37% | 0.009 |
| Married | 54,849,299 57.44% | 63,397,108 59.70% | 118,246,406 58.63% |  |
| **Education** | | | | |
| < High school or less | 29,956,009 31.37% | 42,307,642 39.84% | 72,263,651 35.83% | <0.001 |
| High school - some college | 29,217,024 30.60% | 34,080,233 32.09% | 63,297,257 31.39% |  |
| College graduate or above | 36,308,973 38.03% | 29,806,804 28.07% | 66,115,777 32.78% |  |
| **Family Poverty Income Ratio (PIR)** | | | | |
| Family PIR ≥ 1 | 81,580,794 85.44% | 91,324,121 86.00% | 172,904,915 85.73% | 0.307 |
| Family PIR <1 | 13,901,212 14.56% | 14,870,558 14.00% | 28,771,770 14.27% |  |
| **Insurance** | | | | |
| uninsured | 15,980,829 16.74% | 12,309,492 11.59% | 28,290,321 14.03% | <0.001 |
| Insured | 79,501,177 83.26% | 93,885,188 88.41% | 173,386,364 85.97% |  |
| **Employment** | | | | |
| Unemployed | 26,519,754 27.77% | 46,675,732 43.95% | 73,195,486 36.29% | <0.001 |
| Employed | 68,962,253 72.23% | 59,518,947 56.05% | 128,481,200 63.71% |  |
| **Smoking** | | | | |
| Never Smoker | 60,666,547 63.54% | 56,427,609 53.14% | 117,094,156 58.06% | <0.001 |
| Former Smoker | 18,222,279 19.08% | 31,556,833 29.72% | 49,779,113 24.68% |  |
| Current Smoker | 16,593,180 17.38% | 18,210,236 17.15% | 34,803,416 17.26% |  |
| **Body Mass Index Classification** | | | | |
| Not Overweight/Obese | 36,364,032 38.08% | 20,572,786 19.37% | 56,936,818 28.23% | <0.001 |
| Overweight/Obese | 59,117,974 61.92% | 85,621,893 80.63% | 144,739,867 71.77% |  |
| **Diabetes** | | | | |
| No | 91,637,298 95.97% | 88,728,528 83.55% | 180,365,826 89.43% | <0.001 |
| Yes | 3,844,708  4.03% | 17,466,152 16.45% | 21,310,859 10.57% |  |
| **Chronic Kidney Disease** | | | | |
| No | 94,078,497 98.53% | 101,845,027 95.90% | 195,923,524 97.15% | <0.001 |
| Yes | 1,403,509  1.47% | 4,349,652 4.10% | 5,753,162 2.85% |  |
| Mean ± SD or N (%).  Sleep duration: From the question, “How much sleep do you usually get at night on weekdays or workdays?” Short: ≤6 hours; Recommended: 7–9 hours; Long: >9 hours  All statistics were computed using the weighted sample to account for NHANES’ complex survey design.  P-values are from Kruskal-Wallis tests for continuous variables and chi-square tests for categorical variables.  Analysis restricted to adults with hypertension from the 2011–2023 NHANES sleep duration sample. | | | | |

| **Table S4. Sociodemographic Characteristics by Hypertension Status**  **(2011-2020, Sleep Quality Analysis)** | | | | |
| --- | --- | --- | --- | --- |
|  | **No hypertension** | **Hypertension** | **Total** | **P-value** |
| Sample size | (N=8,975) | (N=12,097) | (N=21,072) |  |
| Population size | 96,447,163 | 106,771,177 | 203,218,340 |  |
| **Age** | | | | |
| Mean (SD) | 40.47 ± (15.03) | 54.30 ± (16.03) | 47.74 ± (17.03) | <0.001 |
| **Race/Ethnicity** | | | | |
| Non-Hispanic White | 64,667,155  67.05% | 73,841,449 69.16% | 138,508,603 68.16% | <0.001 |
| Mexican American/Other Hispanic | 16,698,159  17.31% | 13,453,710 12.60% | 30,151,868 14.84% |  |
| Non-Hispanic Black | 9,047,392  9.38% | 14,190,166 13.29% | 23,237,559 11.43% |  |
| Non-Hispanic Asian | 6,034,457  6.26% | 5,285,853  4.95% | 11,320,310 5.57% |  |
| **Sleep quality** | | | | |
| Good | 22,599,433  23.43% | 36,618,345 34.30% | 59,217,778 29.14% | <0.001 |
| Poor | 73,840,270  76.57% | 70,134,434 65.70% | 143,974,704 70.86% |  |
| **Ever told by doctor have sleep disorder** | | | |  |
| No | 40,961,889  93.15% | 40,596,514 87.24% | 81,558,402 90.11% | <0.001 |
| Yes | 3,010,361  6.85% | 5,938,146  12.76% | 8,948,507 9.89% |  |
| **Sex** | | | | |
| Male | 43,177,044  44.77% | 54,285,946 50.84% | 97,462,990 47.96% | <0.001 |
| Female | 53,270,119  55.23% | 52,485,232 49.16% | 105,755,351 52.04% |  |
| **Marital Status** | | | | |
| Unmarried | 42,146,459  43.70% | 43,530,406 40.77% | 85,676,865 42.16% | 0.002 |
| Married | 54,300,704  56.30% | 63,240,772 59.23% | 117,541,475 57.84% |  |
| **Education** | | | | |
| < High school or less | 30,576,936  31.70% | 42,566,648 39.87% | 73,143,584 35.99% | <0.001 |
| High school - some college | 29,889,710  30.99% | 34,776,767 32.57% | 64,666,477 31.82% |  |
| College graduate or above | 35,980,516  37.31% | 29,427,762 27.56% | 65,408,279 32.19% |  |
| **Family Poverty Income Ratio (PIR)** | | | | |
| Family PIR ≥ 1 | 81,871,938  84.89% | 91,627,491 85.82% | 173,499,429 85.38% | 0.147 |
| Family PIR <1 | 14,575,225  15.11% | 15,143,686 14.18% | 29,718,911 14.62% |  |
| **Insurance** | | | | |
| Uninsured | 17,607,119  18.26% | 13,231,949 12.39% | 30,839,068 15.18% | <0.001 |
| Insured | 78,840,044  81.74% | 93,539,229 87.61% | 172,379,273 84.82% |  |
| **Employment** | | | | |
| Unemployed | 26,591,236  27.57% | 46,786,700 43.82% | 73,377,936 36.11% | <0.001 |
| Employed | 69,855,927  72.43% | 59,984,478 56.18% | 129,840,405 63.89% |  |
| **Smoking** | | | | |
| Never Smoker | 60,353,618  62.58% | 55,837,719 52.30% | 116,191,337 57.18% | <0.001 |
| Former Smoker | 18,549,763  19.23% | 32,166,080 30.13% | 50,715,843 24.96% |  |
| Current Smoker | 17,543,782  18.19% | 18,767,379 17.58% | 36,311,161 17.87% |  |
| **Body Mass Index Classification** | | | | |
| Not Overweight/Obese | 36,995,337  38.36% | 20,803,787 19.48% | 57,799,124 28.44% | <0.001 |
| Overweight/Obese | 59,451,826  61.64% | 85,967,390 80.52% | 145,419,216 71.56% |  |
| **Diabetes** | | | | |
| No | 92,734,887  96.15% | 89,362,598 83.70% | 182,097,485 89.61% | <0.001 |
| Yes | 3,712,276  3.85% | 17,408,580 16.30% | 21,120,856 10.39% |  |
| **Chronic Kidney Disease** | | | | |
| No | 94,958,828  98.46% | 102,426,606 95.93% | 197,385,434 97.13% | <0.001 |
| Yes | 1,488,335  1.54% | 4,344,572  4.07% | 5,832,906 2.87% |  |
| Mean ± SD or N (%).  Sleep quality from the question, “Ever told doctor had trouble sleeping?” Good: No trouble sleeping; Poor: Trouble sleeping  All statistics were computed using the weighted sample to account for NHANES’ complex survey design.  P-values are from Kruskal-Wallis tests for continuous variables and chi-square tests for categorical variables.  Analysis restricted to adults with hypertension from the 2011–2020 NHANES sleep duration sample. | | | | |

| **Table S5. Sociodemographic Characteristics by Hypertension Status**  **(2015-2020, Daytime Sleepiness Analysis)** | | | | |
| --- | --- | --- | --- | --- |
|  | **No hypertension** | **Hypertension** | **Total** | **P-value** |
| Sample size | (N=4,658) | (N=6,838) | (N=11,496) |  |
| Population size | 94,406,542 | 108,199,940 | 202,606,482 |  |
| **Age** | | | | |
| Mean (SD) | 40.92 ± (15.30) | 54.32 ± (16.12) | 48.07 ± (17.10) | <0.001 |
| **Race/Ethnicity** | | | | |
| Non-Hispanic White | 63,359,567 67.11% | 73,463,273 67.90% | 136,822,839 67.53% | <0.001 |
| Mexican American/Other Hispanic | 16,556,769 17.54% | 14,422,864 13.33% | 30,979,633 15.29% |  |
| Non-Hispanic Black | 8,772,184  9.29% | 14,271,632 13.19% | 23,043,816 11.37% |  |
| Non-Hispanic Asian | 5,718,021  6.06% | 6,042,172 5.58% | 11,760,193 5.80% |  |
| **Daytime Sleepiness** | | | | |
| No | 12,930,320 13.70% | 15,093,028 13.96% | 28,023,348 13.84% | 0.705 |
| Mild | 55,913,662 59.23% | 62,789,715 58.07% | 118,703,377 58.61% |  |
| Excessive | 25,562,560 27.08% | 30,242,618 27.97% | 55,805,178 27.55% |  |
| **Ever told by doctor have sleep disorder** | | | | |
| No | 0 .% | 0 .% | 0 .% |  |
| **Sex** | | | | |
| Male | 42,133,568 44.63% | 54,832,268 50.68% | 96,965,836 47.86% | <0.001 |
| Female | 52,272,974 55.37% | 53,367,672 49.32% | 105,640,647 52.14% |  |
| **Marital status** | | | | |
| Unmarried | 38,437,666 40.72% | 42,267,371 39.06% | 80,705,038 39.83% | 0.173 |
| Married | 55,968,875 59.28% | 65,932,569 60.94% | 121,901,444 60.17% |  |
| **Education** | | | | |
| < High school or less | 30,040,348 31.82% | 42,680,201 39.45% | 72,720,549 35.89% | <0.001 |
| High school - some college | 27,995,048 29.65% | 35,601,608 32.90% | 63,596,657 31.39% |  |
| College graduate or above | 36,371,145 38.53% | 29,918,131 27.65% | 66,289,277 32.72% |  |
| **Family Poverty Income Ratio (PIR)** | | | | |
| Family PIR ≥ 1 | 81,844,616 86.69% | 94,450,171 87.29% | 176,294,787 87.01% | 0.436 |
| Family PIR <1 | 12,561,926 13.31% | 13,749,770 12.71% | 26,311,695 12.99% |  |
| **Insurance** | | | | |
| uninsured | 14,028,664 14.86% | 11,173,987 10.33% | 25,202,651 12.44% | <0.001 |
| Insured | 80,377,878 85.14% | 97,025,953 89.67% | 177,403,831 87.56% |  |
| **Employment** | | | | |
| Unemployed | 24,549,722 26.00% | 45,460,373 42.02% | 70,010,095 34.55% | <0.001 |
| Employed | 69,856,820 74.00% | 62,739,567 57.98% | 132,596,387 65.45% |  |
| **Smoking** | | | | |
| Never Smoker | 59,901,841 63.45% | 57,247,688 52.91% | 117,149,529 57.82% | <0.001 |
| Former Smoker | 18,852,053 19.97% | 33,038,217 30.53% | 51,890,270 25.61% |  |
| Current Smoker | 15,652,648 16.58% | 17,914,035 16.56% | 33,566,683 16.57% |  |
| **Body Mass Index Classification** | | | | |
| Not Overweight/Obese | 34,768,364 36.83% | 20,239,236 18.71% | 55,007,600 27.15% | <0.001 |
| Overweight/Obese | 59,638,177 63.17% | 87,960,705 81.29% | 147,598,882 72.85% |  |
| **Diabetes** |  |  |  |  |
| No | 90,316,614 95.67% | 89,757,166 82.95% | 180,073,780 88.88% | <0.001 |
| Yes | 4,089,928  4.33% | 18,442,774 17.05% | 22,532,702 11.12% |  |
| **Chronic Kidney Disease** | | | | |
| No | 92,835,738 98.34% | 103,415,743 95.58% | 196,251,481 96.86% | <0.001 |
| Yes | 1,570,803  1.66% | 4,784,198 4.42% | 6,355,001 3.14% |  |
| Mean ± SD or N (%).  Daytime sleepiness: From the question, “In the past month, how often do you feel overly sleepy during the day?” No: Never (0 times/month); Mild: Rarely (1 time/month) or Sometimes (2–4 times/month); Excessive: Often (5–15 times/month) or Almost Always (16–30 times/month);  All statistics were computed using the weighted sample to account for NHANES’ complex survey design.  P-values are from Kruskal-Wallis tests for continuous variables and chi-square tests for categorical variables.  Analysis restricted to adults with hypertension from the 2015–2020 NHANES sleep duration sample. | | | | |

| **Table S6. Race/Ethnicity, Sleep Outcomes, and Age among US Adults** | | | | | | | |
| --- | --- | --- | --- | --- | --- | --- | --- |
| **Odds Ratio (Confidence Interval)** | | | | | | | |
| **Race/Ethnicity** | **Short** | | | | **Long** | | |
| **Age*** | **19-33** | **34-53** | **54+** | | **19-33** | **34-53** | **54+** |
| **Sleep Duration among persons with hypertension** | | | | | | | |
| **Non-Hispanic White** | 1.00 | 1.00 | 1.00 | | 1.00 | 1.00 | 1.00 |
| **Hispanic**** | 0.96 (0.63,1.46) | 1.02 (0.80,1.30) | **1.28 (1.02,1.61)** | | 0.87 (0.57,1.32) | 0.98 (0.79,1.22) | 0.94 (0.77,1.14) |
| **Non-Hispanic Black** | 1.52 (0.97,2.38) | **1.81 (1.41,2.31)** | **1.98 (1.64,2.39)** | | 1.07 (0.72,1.59) | **1.33 (1.07,1.65)** | 1.00 (0.86,1.17) |
| **Non-Hispanic Asian** | 0.69 (0.38,1.27) | **1.53 (1.08,2.17)** | **1.97 (1.52,2.54)** | | 0.92 (0.57,1.48) | 1.33 (0.97,1.84) | 1.09 (0.86,1.38) |
| **Sleep Duration among persons without hypertension** | | | | | | | |
| **Non-Hispanic White** | 1.00 | 1.00 | 1.00 | | 1.00 | 1.00 | 1.00 |
| **Hispanic**** | 1.16  (0.88,1.53) | 1.12  (0.83,1.51) | **1.65**  **(1.19,2.30)** | | 1.11  (0.87,1.42) | 1.04 (0.83,1.31) | 1.15  (0.88,1.52) |
| **Non-Hispanic Black** | **1.94**  **(1.47,2.56)** | **2.11**  **(1.56,2.86)** | **2.51**  **(1.72,3.66)** | | **1.46**  **(1.13,1.87)** | 1.16 (0.90,1.51) | 1.10  (0.75,1.62) |
| **Non-Hispanic Asian** | 0.91  (0.70,1.18) | 0.99  (0.71,1.38) | **1.79**  **(1.17,2.76)** | | 0.96  (0.69,1.33) | 1.13 (0.86,1.49) | **1.46**  **(1.01,2.12)** |
| **Sleep Quality among persons with hypertension** | | | | | | | |
| **Age*** | **19-33** | | | **34-53** | **54+** | | |
| **Non-Hispanic White** | 1.00 | | | 1.00 | 1.00 | | |
| **Hispanic**** | **1.86 (1.19, 2.91)** | | | **1.73 (1.33,2.25)** | **1.41, (1.16, 1.73)** | | |
| **Non-Hispanic Black** | **1.79 (1.23, 2.62)** | | | **1.42 (1.11,1.82)** | **1.59 (1.36, 1.86)** | | |
| **Non-Hispanic Asian** | **5.40 (2.87, 10.19)** | | | **2.59(1.92, 3.48)** | **2.15 (1.64, 2.82)** | | |
| **Sleep Quality among persons without hypertension** | | | | | | | |
| **Age*** | **19-33** | | | **34-53** | **54+** | | |
| **Non-Hispanic White** | 1.00 | | | 1.00 | 1.00 | | |
| **Hispanic**** | **1.70 (1.24, 2.30)** | | | **1.69 (1.28,2.22)** | 1.31(0.96, 1.79) | | |
| **Non-Hispanic Black** | **1.97 (1.45, 2.69)** | | | **1.64 (1.18,2.27)** | **1.66 (1.19, 2.31)** | | |
| **Non-Hispanic Asian** | **1.89 (1.34, 2.66)** | | | **2.30 (1.68,3.15)** | **2.72 (1.62, 4.57)** | | |
| Reference sleep duration = Recommended sleep duration (7–9 hours per night), versus short sleep (≤6 hours) and long sleep (≥10 hours), based on self-reported usual sleep on weekdays/workdays. Reference sleep quality = Good sleep quality, defined as never being told by a doctor or other health professional that they had trouble sleeping, versus poor sleep quality, defined as ever being told they had trouble sleeping. Race/Ethnicity categories: ** Hispanic includes Mexican American and Other Hispanic adults (combined); Non-Hispanic White, Non-Hispanic Black, and Non-Hispanic Asian were analyzed separately as mutually exclusive groups. All models compare racial/ethnic groups to Non-Hispanic White adults within each age and hypertension status stratum, accounting for NHANES complex sampling and survey weights. | | | | | | | |

**Figures**


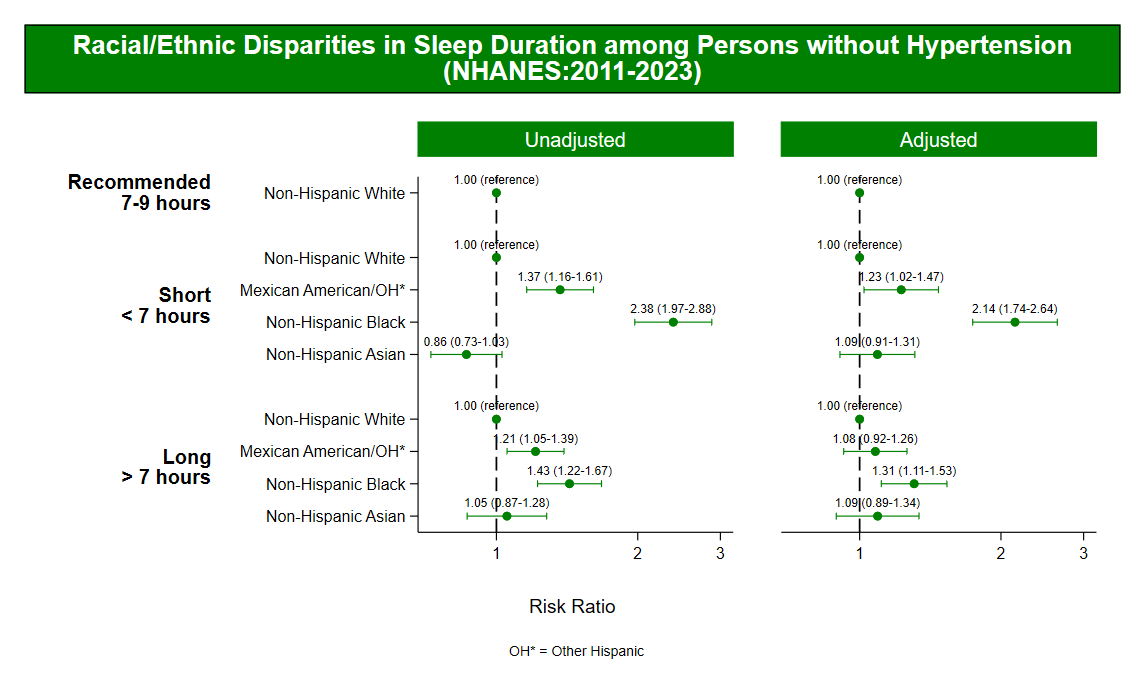


**Figure S1. Adjusted odds ratios for short and long sleep duration by race and ethnicity among U.S. adults without hypertension (2011–2023).**

Forest plot depicting associations between race/ethnicity and sleep duration (Recommended [7–9 hours], Short [≤6 hours], Long [>9 hours]) among adults without hypertension. Non-Hispanic White adults serve as the reference group. Models adjusted for age, sex, education, poverty income ratio, insurance, marital status, employment, smoking, diabetes, body mass index, and CKD.


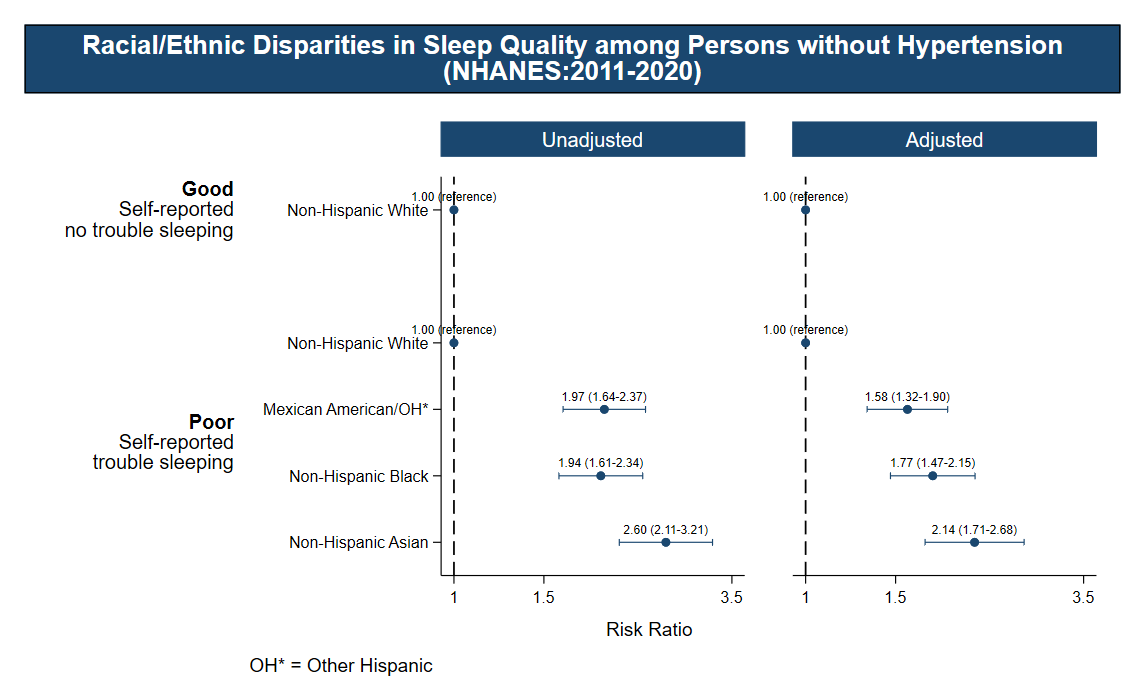


**Figure S2. Adjusted odds ratios for poor sleep quality by race and ethnicity among U.S. adults without hypertension (2011–2020).**

Forest plot displaying associations between race/ethnicity and sleep quality among adults without hypertension. Poor sleep is defined as self-reported trouble sleeping. Reference group is non-Hispanic White adults. Models adjusted for age, sex, education, poverty income ratio, insurance, marital status, employment, smoking, diabetes, body mass index, and CKD.


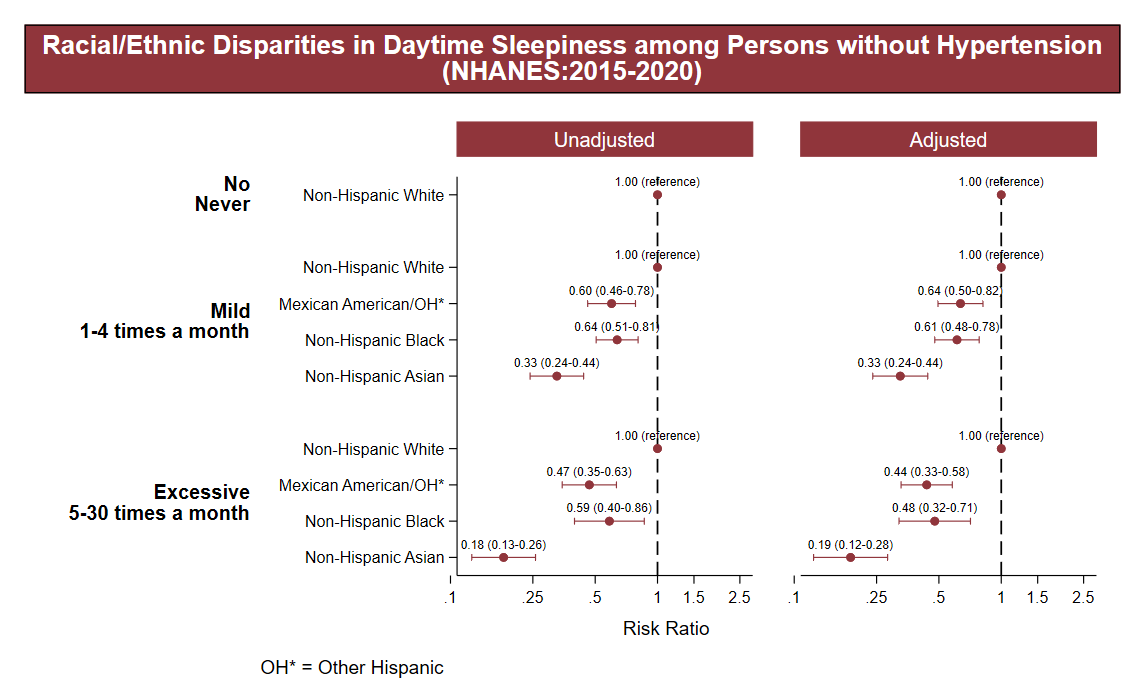
.

**Figure S3. Adjusted odds ratios for excessive daytime sleepiness by race and ethnicity among U.S. adults without hypertension (2015–2020).**

Forest plot of associations between race/ethnicity and levels of daytime sleepiness (No, Mild, Excessive) among adults without hypertension. Non-Hispanic White adults serve as the reference group. Models adjusted for age, sex, education, poverty income ratio, insurance, marital status, employment, smoking, diabetes, body mass index, and CKD.
